# Pseudohypoxic brain swelling following cerebrospinal fluid leakage: a case report on rapid identification and multidisciplinary management

**DOI:** 10.1093/jscr/rjae520

**Published:** 2024-08-19

**Authors:** Lennard M Wurm, Lukas Neuhaus, Golschan Aspargur, Stefan Angemair, Dominik Laue

**Affiliations:** Department of Traumatology and Reconstructive Surgery, Charité – Universitätsmedizin Berlin, Freie Universität Berlin, Humboldt-Universität zu Berlin and Berlin Institute of Health, 10117 Berlin, Germany; Department of Traumatology and Reconstructive Surgery, Charité – Universitätsmedizin Berlin, Freie Universität Berlin, Humboldt-Universität zu Berlin and Berlin Institute of Health, 10117 Berlin, Germany; Department of Anesthesiology and Intensive Care Medicine, Charité – Universitätsmedizin Berlin, Freie Universität Berlin, Humboldt-Universität zu Berlin and Berlin Institute of Health, 10117 Berlin, Germany; Department of Anesthesiology and Intensive Care Medicine, Charité – Universitätsmedizin Berlin, Freie Universität Berlin, Humboldt-Universität zu Berlin and Berlin Institute of Health, 10117 Berlin, Germany; Department of Traumatology and Reconstructive Surgery, Charité – Universitätsmedizin Berlin, Freie Universität Berlin, Humboldt-Universität zu Berlin and Berlin Institute of Health, 10117 Berlin, Germany

**Keywords:** pseudohypoxic brain swelling, cerebrospinal fluid leak, spine surgery

## Abstract

This report delineates the intricate diagnostic journey and therapeutic conundrum presented by a 61-year-old male who exhibited atypical neurological deterioration shortly after lumbar fusion surgery, manifesting clinical and radiological features suggestive of pseudohypoxic encephalopathy, an entity characterized by symptoms mimicking cerebral hypoxia in the absence of a discernible hypoxic insult. Following an initially unremarkable recovery from an elaborate spinal surgery, the patient’s postoperative condition was confounded by a perplexing decline in consciousness, unresponsive to conventional therapeutic interventions and devoid of clear etiological indicators on standard neuroimaging. The subsequent diagnostic odyssey unraveled a cerebrospinal fluid leak as the putative reason, positing a nuanced clinical paradigm wherein the cerebrospinal fluid leak engendered a state mimicking pseudohypoxic brain swelling. This report underscores the clinical challenges and emphasizes the need for an astute diagnostic approach in postoperative patients with unexplained neurological symptoms advocating for a comprehensive evaluation to identify underlying cerebrospinal fluid leaks and mitigate potential morbidity.

## Introduction

Pseudohypoxic brain swelling is a rare but serious condition characterized by brain swelling and damage that mimics the effects of hypoxia despite adequate oxygen levels in the blood. This condition can occur following cerebrospinal fluid (CSF) leakage, leading to a complex clinical picture that requires prompt diagnosis and management to prevent irreversible brain damage. This case report outlines one of few but evolving unique instances of pseudohypoxic brain swelling following a CSF leak in a 61-year-old male patient undergoing lumbar fusion surgery, as it has only been rarely reported in the past. The evidence strongly suggests that this new disease entity is an increasingly described result of uncomplicated surgery [1–8].

## Case description

**Figure 1:**
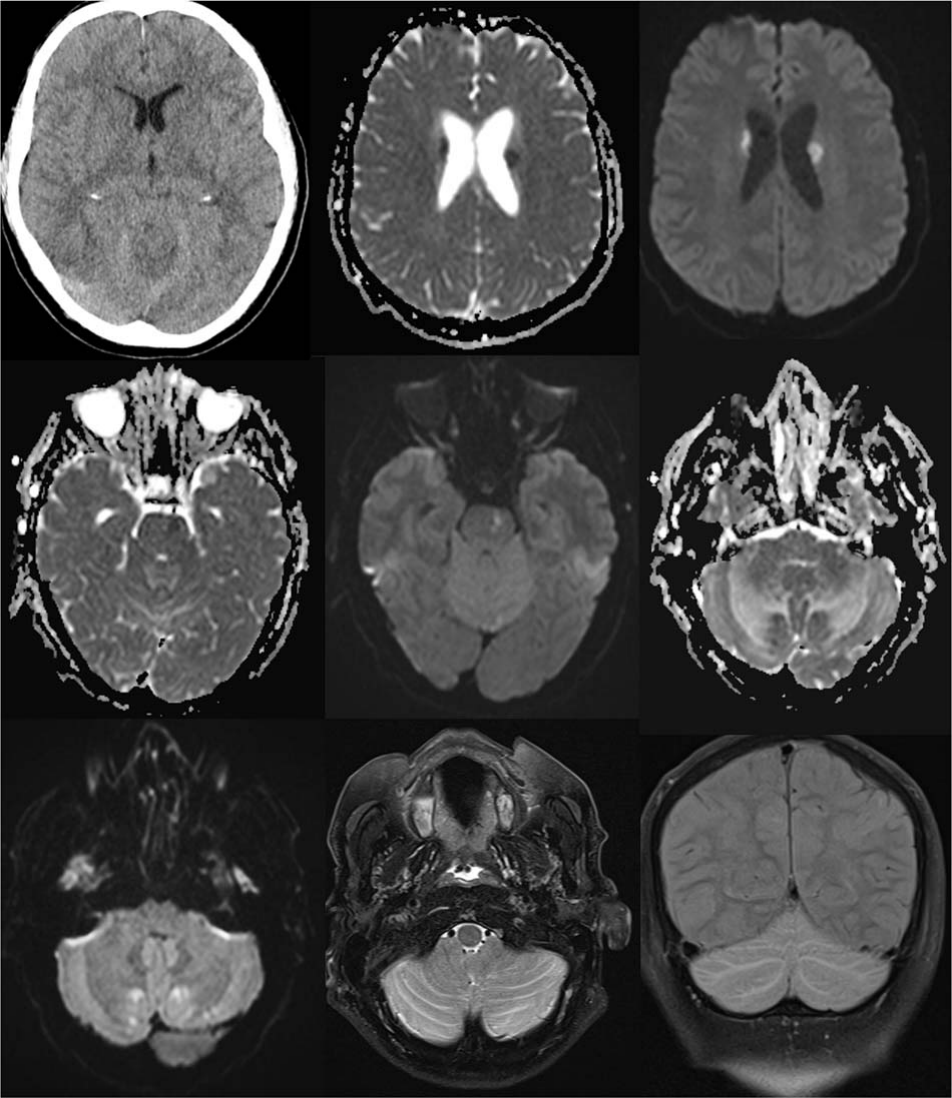
Comprehensive computer tomographic (CT) and magnetic resonance imaging (MRI) of pseudohypoxia brain damage due to CSF loss; from left to right, top to bottom: (1) CT axial view showing general brain structure and pathology; (2 + 3) diffusion-weighted imaging (DWI) and apparent diffusion coefficient (ADC) maps of the basal ganglia, and (4 + 5) DWI and ADC images of the pons; (6 + 7) DWI and ADC images of the cerebellum; (8) T2 Turbo Spin Echo with fat saturation (TSE FS) axial view of the cerebellum; (9) Sagittal T2 turbo inversion recovery magnitude coronal view depicting tonsillar descent with cerebellar edema, and these images illustrate the intricate brain changes associated with pseudohypoxia damage following CSF leakage.

A 61-year-old male was electively admitted for surgical management of adjacent segment instability in the lumbar level L2/3 after fusion of the segments L3-S1 in the past. The surgical procedure proceeded intra-operatively without complications. However, rapid vigilance reduction was noted in the recovery room. Initial assessments ruled out opioid overdose and non-convulsive seizure with the administration of Naloxone and benzodiazepines, leaving the cause of reduced vigilance unclear. Protective intubation was performed in the recovery room, and cranial CT (cCT) scans revealed effaced sulcal relief and hypodense basal ganglia, suggesting the onset of hypoxic brain damage (as seen in Fig. 1).

**Figure 2:**
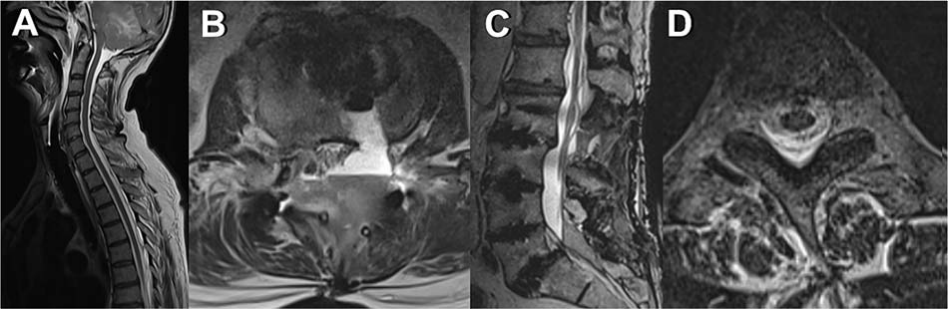
MRI scans depicting various manifestations of liquor leakage; (A) TSE sagittal view of the cervical spine, illustrating fluid distribution and spinal alignment; (B) axial view at the level of the third thoracic vertebra (T3), showing detailed cross-sectional anatomy, and (C) *T*_2_-weighted sagittal view, highlighting the spinal cord and surrounding structures; (D) axial view at the level of the second and third lumbar vertebrae (L2/3), providing insights into lower spinal segments.

The patient was admitted to the intensive care unit, cardiopulmonary stable but without awakening response after sedation cessation. Cranial MRI revealed basal subarachnoid hemorrhage, significant cerebellar vasogenic edema, swelling of the posterior cranial fossa, tonsillar descent, and ventricular compression, indicative of pseudohypoxic brain swelling due to CSF loss (as seen in Fig. 1). The surgical drainages were delivering no signs of CSF loss. An external ventricular drain (EVD) was placed for brain pressure compensation showing a clear appearing CSF with an opening pressure over 20 mmHg. A conservative intracranial pressure therapy with mannitol and deepening of sedation was initiated. Subsequent CT imaging showed progressing global brain edema and cerebellar and basal ganglia infarcts. The following cranial and spinal MR Imaging showed an extensive CSF leakage with wide epidural spread, necessitating revision surgery to cover a 4-mm dural tear at the lumbar level L2/3 by stitching over the dura and applying a Tachosil sponge (as seen in Fig. 2).

Gradual successful weaning, increasing responsiveness, and MRI control showed demarcation of infarcts in the striatum, pons, cerebellum, and reduced brain edema, leading to the EVD being removed. Upon extubation, the patient was clear in orientation to place and person, partially oriented in time with dysarthrophonia, dysphagia, right-sided hemiparesis, and dysmetria on the right. He rapidly gained orientation with regressive right-sided hemiparesis. Following transfer to the orthopedic regular ward, the patient underwent physiotherapy and nutritional buildup under speech therapy. Treatment for pneumonia with Piperacillin/Tazobactam and *Clostridium difficile* diarrhea with oral Vancomycin was necessary. After early neurorehabilitation on the orthopedic ward the patient achieved a remarkable recovery with remaining issues including slight dysphagia, latent right-sided hemiparesis and dysmetria on the right. The patient was discharged to neurorehabilitation 2 months after surgery.

## Discussion

This case highlights the complexities of diagnosing and managing pseudohypoxic brain swelling following CSF leakage. The initial symptoms of reduced vigilance without clear etiology underscore the importance of considering CSF leak and subsequent pseudohypoxic brain swelling in differential diagnoses, particularly postoperatively.

The successful management involved rapid diagnostic imaging, surgical intervention to address CSF leak, and intensive supportive care including measures to manage cerebral edema and complications arising from prolonged intensive care. In conclusion, this case underscores the imperative to focus on prompt MR imaging of the spine and the surgically treated segment to confirm or rule out differential diagnoses, thereby facilitating timely and effective clinical interventions.

## Conclusion

Pseudohypoxic brain damage following CSF leakage presents a challenging diagnostic and therapeutic scenario. This case underscores the importance of a high index of suspicion for CSF leak in patients exhibiting unexplained postoperative neurological decline. Early intervention, including surgical repair of the CSF leak and management of resultant brain edema, is critical to improving outcomes. Moreover, this case emphasizes the potential for significant recovery with comprehensive rehabilitation, highlighting the resilience of the human brain and the importance of multidisciplinary care in managing complex neurological conditions.

## Data Availability

Data generated during and/or analyzed during the current study are available from the corresponding author on reasonable request.
